# Testing the Use of Static Chamber Boxes to Monitor Greenhouse Gas Emissions from Wood Chip Storage Heaps

**DOI:** 10.1007/s12155-016-9800-9

**Published:** 2016-11-09

**Authors:** Carly Whittaker, Nicola E. Yates, Stephen J. Powers, Neil Donovan, Tom Misselbrook, Ian Shield

**Affiliations:** 1 Department of Agro-Ecology, Rothamsted Research, Harpenden, Hertfordshire AL5 2JQ UK; 2 Department of Computational and Systems Biology, Rothamsted Research, Harpenden, Hertfordshire AL5 2JQ UK; 3 Department of Sustainable Soils and Grassland Systems, Rothamsted Research North Wyke, Okehampton, Devon EX20 2SB UK

**Keywords:** Bioenergy, Storage, Supply chain, Short rotation coppice willow, Methane

## Abstract

This study explores the use of static chamber boxes to detect whether there are fugitive emissions of greenhouse gases (GHGs) from a willow chip storage heap. The results from the boxes were compared with those from 3-m stainless steel probes inserted into the core of the heap horizontally and vertically at intervals. The results from probes showed that there were increases of carbon dioxide (CO_2_) concentrations in the heap over the first 10 days after heap establishment, which were correlated with a temperature rise to 60 °C. As the CO_2_ declined, there was a small peak in methane (CH_4_) concentration in probes orientated vertically in the heap. Static chambers positioned at the apex of the heap detected some CO_2_ fluxes as seen in the probes; however, the quantities were small and random in nature. A small (maximum 5 ppm) flux in CH_4_ occurred at the same time as the probe concentrations peaked. Overall, the static chamber method was not effective in monitoring fluxes from the heap as there was evidence that gases could enter and leave around the edges of the chambers during the course of the experiment. In general, the use of standard (25 cm high) static chambers for monitoring fluxes from wood chip heaps is not recommended.

**Electronic supplementary material:**

The online version of this article (doi:10.1007/s12155-016-9800-9) contains supplementary material, which is available to authorized users.

## Introduction

Short rotation coppice (SRC) willow is conventionally cut in late winter/early spring, prior to bud burst in order to maximise potential re-growth in the following growing season [1]. As the crop is harvested in large quantities and at a moisture content (MC) of about 50 %, it is beneficial to simultaneously dry and store the material in order to provide a suitable quality fuel at the time of demand [2]. Forced drying and protected storage can be expensive, so a solution is to pile the freshly cut wood chips into heaps outside, where they can dry from exposure to direct sunlight and natural ventilation [3].

Many studies report a rapid increase in temperature to over 60 °C after wood chip heaps are established, which is an indication of extensive microbial activity, known as the mesophilic phase [4] observed in composting processes [5]. This is due to the microbial degradation of readily available soluble carbohydrates in the wood [6] and has been demonstrated to coincide with a peak in carbon dioxide (CO_2_) concentration in both pine sawdust [6] and willow chip heaps [7]. This breakdown process can involve the rapid depletion of oxygen so that anaerobic conditions prevail in the core parts of the heap [4, 8], which may lead to the generation of methane (CH_4_) [9]. Little is known about the extent to which this occurs in wood chip storage heaps [10]; however, a recent study discovered a peak in CH_4_ concentration using probes embedded in a willow chip heap built on grassland [7]. Interestingly, the CH_4_ peak coincided with a drop in CO_2_ concentration. The observations suggested that, after an active period of aerobic decomposition in the first 2 months of storage, the conditions in the heap became anaerobic.

Sampling methods with gas probes suffer from a drawback of not being able to detect the rate at which the gases leave the heap [10], which, if substantial, could compromise the GHG emission savings from utilising wood chip stored in this way. A recent study showed that if 1 % of the carbon in the stored biomass left the wood chip heap, the GHG emissions per megawatt hour (MWh, of heat) would triple [11]. In biological systems, CH_4_ can be oxidised by methanotrophic bacteria to water and CO_2_ [12], so concentrations recorded within the centre of the heap do not prove that fugitive emissions occur. Some studies have attempted to quantify this in compost by using static flux chambers on the periphery of storage heaps [4]. This method works by measuring the build-up of concentrations of gases in the headspace of the chambers to calculate a net flux from the substrate being measured. This method has conventionally been carried out to measure fluxes of nitrous oxide from soils [13]. The method has not been performed on wood chip heaps; therefore, the aim of this study was to compare the temporal GHG emission profile of the SRC willow chip heap from using probes to that from using a series of static flux chambers.

## Methods

### Wood Chip Heap Construction

Approximately 74 t of fresh SRC willow, with a MC of 56 %, was harvested from two adjacent areas (coordinates 52.012854, −0.598906). The willow sites were planted in 2009, cut back in 2010 and were previously harvested in winter 2011–2012, after 2 years of growth. The areas were planted with breeding material from a *Salix viminalis* x *Salix schwerinii* cross with both sites previously cropped in an arable rotation. The SRC willow was treated with a residual herbicide (aminotriazole) and 60 kg/ha nitrogen in spring 2012.

The crop was harvested on 4 March 2015, before bud burst using a Claas forager harvester with a Coppice Resources Ltd. (Retford, UK) header. The harvester simultaneously harvests and chips the crop and was set up to produce a chip with an average length of 30 mm. The bulk density and carbon and nitrogen contents of the fresh wood chip are detailed in Table 1. The material was immediately transported to a site on a level field near Woburn Farm, Husborne Crawley, Bedfordshire, historically long-term grassland where the heap was built. The heap was formed by unloading the chips onto the ground and piling them up into a heap using a tractor with a front mounted loader and bucket avoiding contamination of the heap with grass and soil. The completed heap was approximately 19 m long, 7 m wide and 3 m high and was built in a precise south-westerly to north-easterly orientation, as a dominant (54 %) south-westerly wind direction was recorded at the site in the previous year (2014). Therefore, the heap was built with one end facing the prevailing winds.

**Table 1:** Characteristics of the stored material

Parameter	Units	Freshly harvested willow chip
Moisture content	% wb	56.4
Ash content	% db	1.7
C content	% db	49.0
N content	% db	0.3
Bulk density	kg/m^3^	447

*wb* wet basis, *db* dry basis

### Temperature Records

**Fig. 1:**
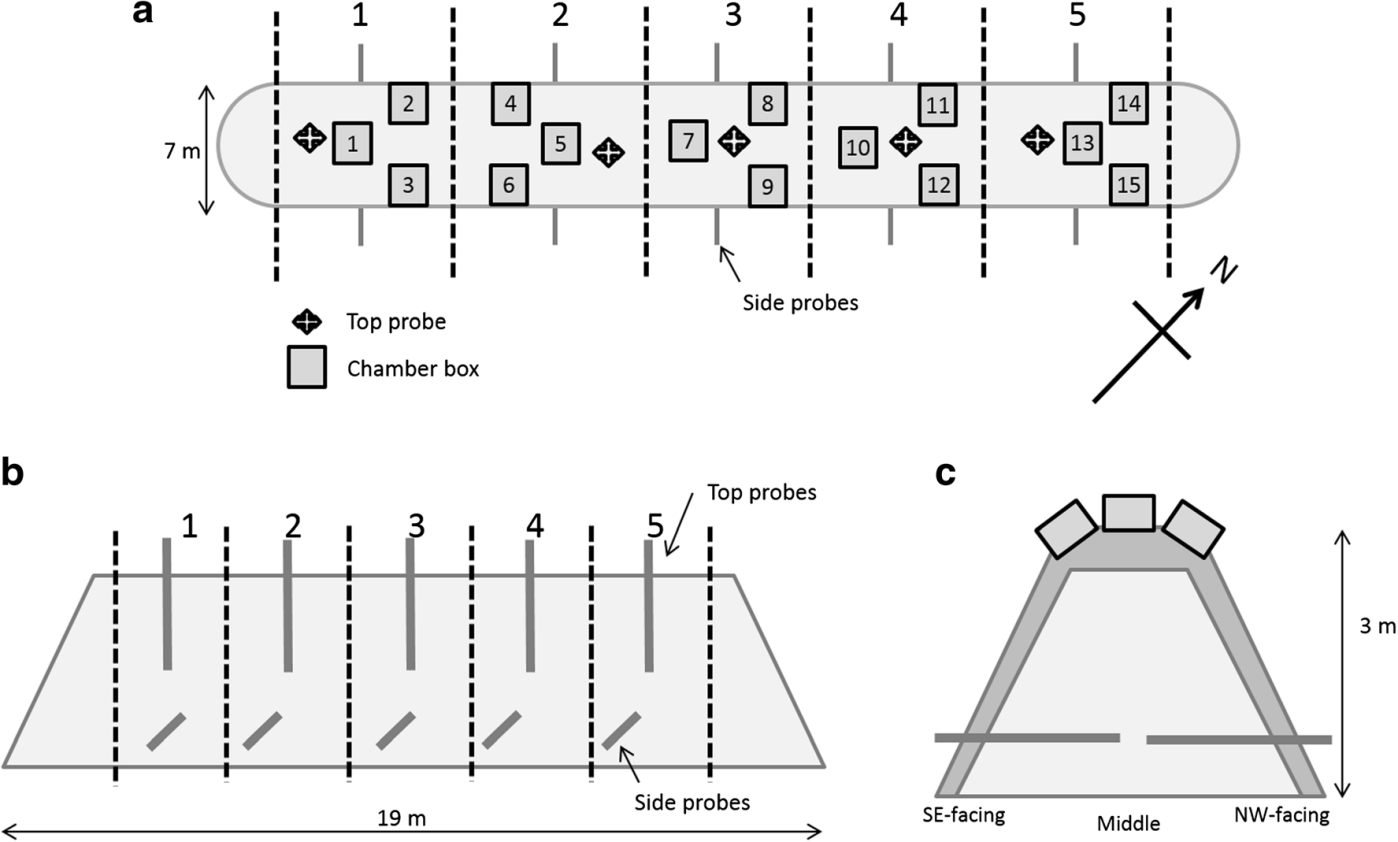
Diagram of the design of the wood chip heap. **a** Bird’s eye view of the heap with location of zones (*1–5*), boxes and top and side probes, **b** side profile with top and side probes and **c** cross section of heap with flux box positioning and side and top probes

The heap was divided into five zones (Fig. 1a, b) for the purposes of analysis. A temperature recorder (Log Tag® Model Trix-8, LogTag Recorders Ltd., Auckland, New Zealand) was added to bagged (netted bags) samples of chip. Three of these bags were placed in the core area of each zone so that they were at least 2–3 m under the surface, and one was placed in the top of the stack (Fig. 1c). Ambient weather records were taken using the Woburn weather station situated 500 m from the heap.

### Greenhouse Gas Emission Sampling

Stainless steel probes were used to take gas samples from the five zones of the heap (Fig. 1). A probe was inserted on the SE and NW sides as well as at the top (middle) in each zone, giving 15 probes altogether, to sample from a horizontal or vertical depth of 2 m. Greenhouse gas concentrations from the probes (CO_2_, CH_4_ and N_2_O) were measured twice a week for the first 3 months, then weekly according to the GHG gas profile. The final measurement was taken on 23 June 2015. On each occasion, five samples were also taken from the ambient air.

Static flux chambers were installed around the apex of the heap (Fig. 1) so that gas flux could be sampled using the method described by Anderson et al. [14]. The mentioned study was performed on compost heaps and found that flux chambers positioned at the apex of the heap accounted for 85–100 % of the total gaseous flux [14]. This may be due to the ‘chimney effect’ caused by flow of heat and gases according to conduction and condensation in the apex, a phenomenon observed in wood storage heaps [2].

A total of 15 static flux chambers were used: one directly on the top of the heap (middle) and one on each side of the top (SE and NW) of each zone. The flux chambers were 40 × 40 × 25 cm in dimension and were inserted into the heap to a depth of approximately 15 cm leaving 10 cm protruding. After allowing the boxes to settle for 2 days, gas sampling was performed according to the methodology described in Collier et al. [13]. The chamber lids were fitted into place, and then, samples were taken at 0, 20, 40 and 60 min. Samples were taken from the probes and flux chambers on the same day and were taken every 3–4 days for the first month and weekly thereafter.

All gas samples were transferred to 22-ml pre-evacuated glass vials for transport and storage prior to gas concentration analysis. The gas samples were analysed for CO_2_, CH_4_ and N_2_O concentration using a PerkinElmer Clarus 500 Gas Chromatograph (GC) linked to a PerkinElmer TurboMartrix 110 headspace autosampler (PerkinElmer, Waltham, MA, USA). The GC was fitted with a flame ionisation detector (FID) housing a methaniser for the measurement of CO_2_ and CH_4_ concentration and an electron capture detector (ECD) for the measurement of N_2_O concentration. Each gas sample was split between two identical PerkinElmer megabore capillary Elite PLOT Q columns for delivery to the two detection systems. The FID was set at 350 °C, whilst the ECD was set at 300 °C. A bracketed calibration employing five gas standards (mixtures of known amounts of CH_4_, CO_2_ and N_2_O in synthetic air) was used with each batch of samples, and check samples of known concentration were included at regular intervals within each sample run.

Gas flux (*F*, mg/m^2^/h) from the chamber measurements on each sampling occasion was determined from the linear increase in headspace concentration over the 60-min sampling period according to

$$ F=\rho \frac{V}{A}\frac{\varDelta c}{\varDelta t}\frac{273}{\left(T+273\right)} $$

where *ρ* is the gas density (mg/m^3^), *V* the chamber headspace volume (m^3^), *A* is the surface area covered by the chamber (m^2^), Δ*c*/Δ*t* is the increase in headspace gas concentration *c* over time *t* (ppm/h) and *T* is the chamber air temperature (°C). If the increase in headspace concentration was non-linear over the 60-min period (i.e. if increase in headspace concentration was inhibiting emission), then flux was derived using the 0–60-min samples.

### Statistical Analysis

For the data derived from the gas probes, the method of residual maximum likelihood (REML) was used to fit a linear mixed model to each measurement response, consisting of random terms for the design used (zones, probes within zones and time points within probes) and fixed terms for the treatment terms to be tested (air versus heap comparison, location of probe (SE, NW, middle) and time of measurement) using approximate *F* tests. The non-independence of measurements taken from the same probes over time was accounted for by imposing a power model structure, as a further random term, for time within probes. This could be fitted assuming a common variance structure over all time points and then assuming a different variance at each time point to test (using change in model deviance distributed as chi-squared) for heterogeneity of variance over time. Finally, the significance of spline terms was tested, again using change in model deviance, to assess the extent of curvature over time as a whole and then separately for either the air versus heap categorisation or the full set of four treatments (air, SE, NW and middle).

A similar modelling procedure was used for the flux data, albeit now without a comparison to the air being required and with repeated measures now being from the chamber boxes rather than the probes. A natural log (to base *e*) transformation was used for the data from the gas probes to ensure a normal distribution and to account for heterogeneity of variance over the locations; no transformation was required for the CH_4_ flux data, but the log transformation was used for CO_2_ flux along with a small adjustment (0.008) to account for negative values.

Temperature data from the recorders located in bagged samples were modelled in the same way as the gas data to pull out the trends over time in the crust and core regions of the heap.

The GenStat (17th edition, © VSN International Ltd., Hemel Hempstead, UK) statistical package was used for all analyses.

## Results

### Temperature Profile and Moisture Content

**Fig. 2:**
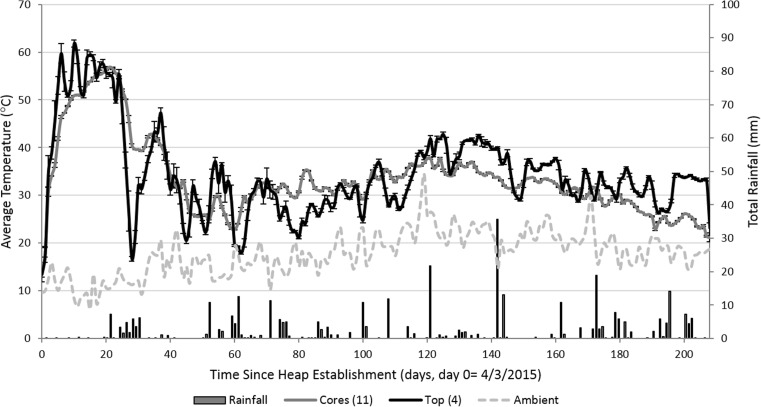
Average daily temperature from temperature recorders placed in netted bags located in the core and top of the heap alongside ambient temperature (*left y*-axis) and total rainfall (*right y*-axis). *Error bars* on the temperature readings show standard error

The records retrieved from the Log Tag® recorders placed in the netted bags are shown alongside ambient temperatures and rainfall (Fig. 2). During the course of the experiment, the site received 354 mm of rainfall. During heap destruction, samples of the MC of the core and crust of the heap were recorded. The MC of the chip from the core was relatively consistent over the five zones (average 38 %, SE 1.4) and was significantly drier than that from the outer crust, which averaged 59 %, SE 2.4 (*F* = 91.20 on 1 and 42 df, *p* < 0.001). The crust showed large variations in MC and was around 30 cm in depth throughout the stack. The top of the stack was the wettest part (MC 72.4 %), indicating the same chimney effect observed in other studies of stacked biomass, with transition of water upwards where it cools and condenses [2, 5, 14, 15]. Therefore, the core of the stack dried from 55 to 38 %, and there was evidence of a convection current in the heap. Based on the change in mass of the heap and the standard error of measured MC before and after storage, a total heap dry matter loss of between 5 and 6 t or 19.8 and 22.6 % was estimated.

The temperature results are based on 19 Log Tag® recorders that were retrieved from the heap: 11 from the core of the heap and 4 from the top (four were damaged). Average temperatures at the top of the heap peaked at 62 °C after 10 days, whereas the core warmed more slowly and reached 58 °C after 20 days. High average temperatures of over 50 °C were recorded until day 28 in all locations in the heap, after which there was a decline in temperature, particularly at the top of the heap. Indeed, over the course of the experiment, the temperature at the top appeared to fluctuate more than that in the core. In some instances, drops in temperatures at the top appeared to be associated with low ambient temperatures and rainfall events, particularly on days 21, 52, 71, 99, 110 and 142.

After the self-heating phase, the heap cooled to around 30 °C, which it remained until the end of the experiment. Overall, each zone followed a similar pattern in temperature profile, and in general, the top of the heap was most often warmer than the core until day 70 when a crossover occurred. However, after 120 days (during the summer months), the top was once again generally warmer than the core.

Overall, there was no significant (*p* < 0.05, *χ* ^2^ test) correlation over time for the temperature data, meaning that having accounted for variance due to the overall non-linear trends of heating and cooling, the remaining variance in heap temperature occurred independent of time.

### Greenhouse Gas Losses

The following two sub-sections describe the concentrations of GHGs measured in the heap via the probes and the fluxes measured using the static chamber boxes. Throughout the experiment, N_2_O was detected in quantities similar to ambient levels at all locations in the heap and was thus excluded from the analysis.

#### Heap Concentrations

**Fig. 3:**
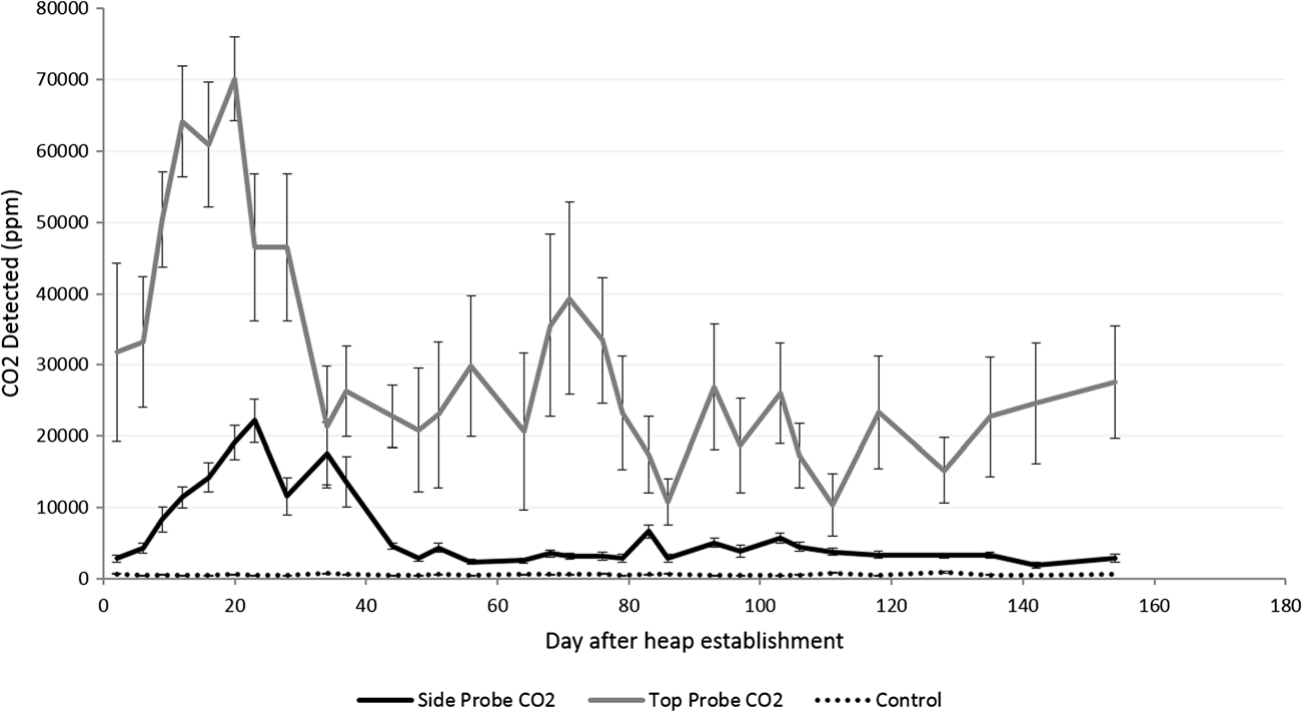
Means and standard errors of the concentration of CO_2_ in top and side probes

**Fig. 4:**
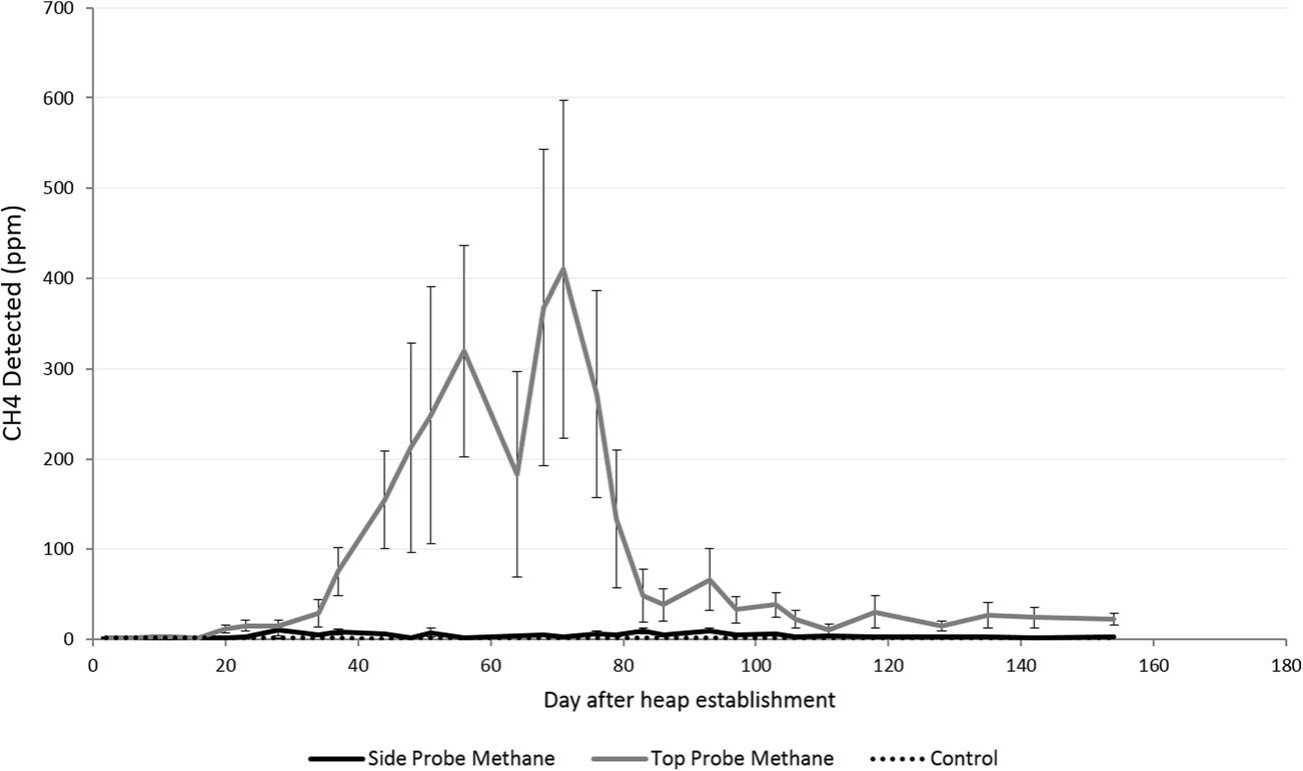
Means and standard errors of the concentration of CH_4_ in top and side probes

The CO_2_ and CH_4_ concentration profiles detected within the two heaps were significantly different from the ambient samples (*p* < 0.05, *F* tests) and showed significant curvature over time which was specific to the SE and NW side probes and the top probe (*p* < 0.001, *χ* ^2^ tests for spline terms). The GHG emission profile taken from the air, side and top probes for both gases is shown in Figs. 3 and 4 for CO_2_ and CH_4_, respectively. The trends recognised by the spline terms for CO_2_ and CH_4_ are shown in the Supplementary data 1a and b, respectively. In all instances, higher concentrations of CO_2_ and CH_4_ were detected in the top probes compared to the side probes.

The results show an initial peak in the CO_2_ concentrations in both top (c. 70,000 ppm) and side (c. 20,000 ppm) probes around days 20–23 (Fig. 3). After this, there was a steady decline in CO_2_ concentrations in the NW and SE side probes; however, measurements from the top probes were generally more erratic, with an apparent second peak (c. 40,000 ppm) on day 71. The results show that greatest variance was between days 28 and 44. The CH_4_ concentration began to increase steadily after day 20 (Fig. 4), while the CO_2_ concentrations were at their maximum. This continued until they peaked at 400 ppm on day 71 before a rapid decline. The results show that greatest variance in CH_4_ concentrations was between days 34 and 64, at a time when the internal temperature was quite variable too. This was only observed in the top probes, which gave significantly higher CH_4_ concentrations than the side probes (*F* = 71.7 on 2 and 71 df, *p* < 0.001, *F* test), for which CH_4_ did not rise much higher than 2–3 ppm above ambient during the course of the experiment.

**Fig. 5:**
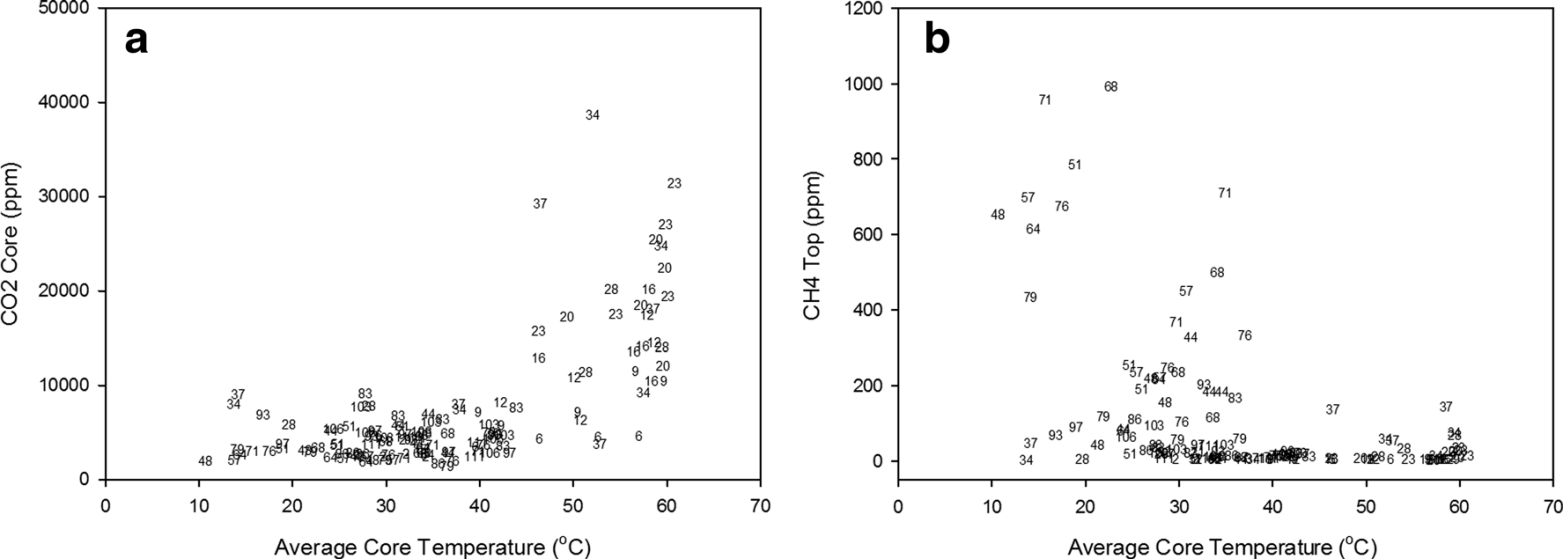
Correlations detected between CO_2_ (**a**) and CH_4_ (**b**) and average core temperature. The plotted points are labelled as numbers, being the days when the heaps were sampled

**Fig. 6:**
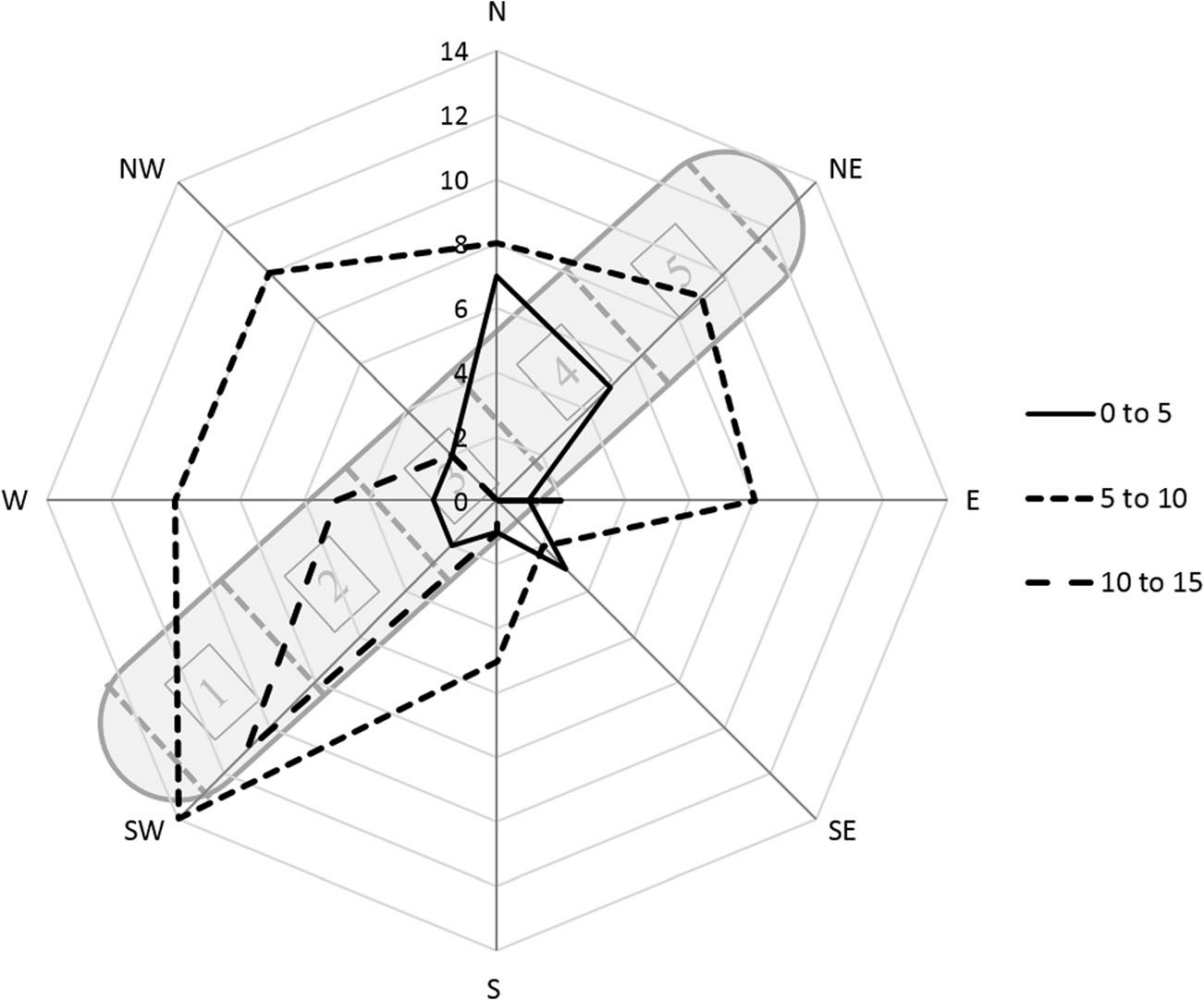
Frequency of days with given wind direction and speed (m/s) during the course of the sampling period, superimposed on the relative positioning of the heap (NE to SW)

The correlation between heap temperature and CO_2_ concentration from the core was positive and greater than 0.50, indicating an important correlation (*r* = 0.663, *n* = 130, *p* < 0.001, *F* test, Fig. 5a), which was particularly due to days with high core temperatures (Fig. 2). The temperature of the heap correlated significantly (*p* < 0.05, *F* test) with the CH_4_ concentrations recorded from the top probe, but the correlation was not greater than 0.50 in magnitude. The data suggests that CH_4_ concentrations were higher during cooler temperatures. This negative correlation (for example, *r* = −0.469, *n* = 129, *p* < 0.001, *F* test in Fig. 4b) corresponds to the theory that anaerobic decomposition occurs at lower temperatures than aerobic decomposition. In Fig. 5b, the eight points at the top left in the scatter plot are from zone 4 for samples taken between days 44 and 76. Although the negative correlation would clearly persist without them, it would certainly be far less strong. During these days, the wind direction was predominately (63 %) from the SW direction, with an average speed of 4.4 m/s, so zone 4 may have been particularly sheltered during this time (Fig. 6).

For both gases, significantly higher concentrations were detected in the top probes. These were sealed between sampling, so it is possible that there was some accumulation of gases inside the probes. The probes showed a decline in gas concentrations after the observed peak, suggesting that if gases accumulated in the probes, they were able to escape later.

#### Static Flux Chambers

**Fig. 7:**
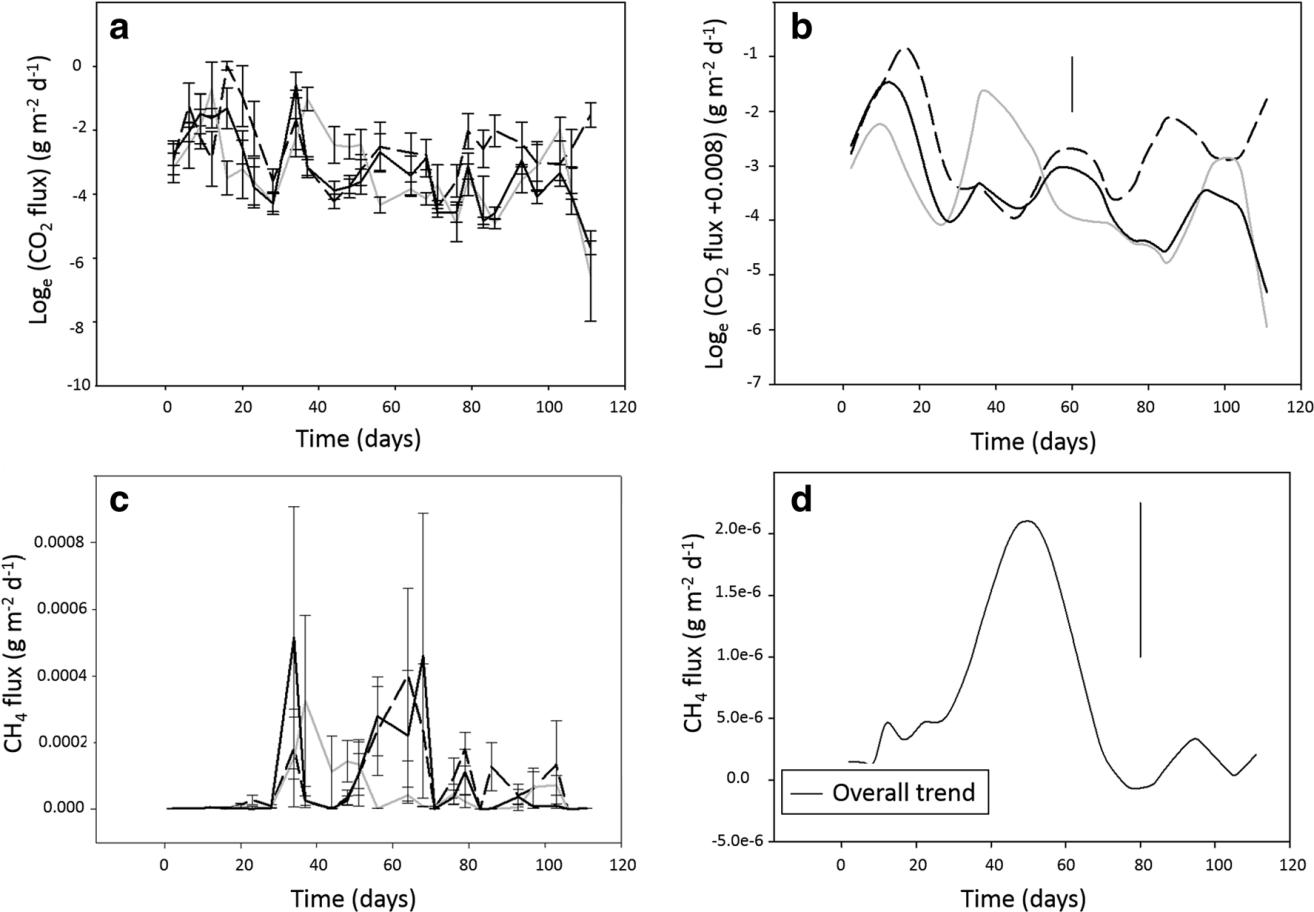
Quantity of CO_2_ (**a**, **b**) and CH_4_ (**c**, **d**) in the flux boxes on the SE and NW (top sides) and directly on the top of the heap. Parts **a** and **b** show means and standard errors of the gases (on the natural log scale for CO_2_); **b** and **d** show the corresponding modelled trend recognised by the spline terms (see Statistical Analysis and Static Flux Chambers sections)

During the course of the experiment, a total of 400 samples of flux were taken. During these sampling periods, 27 and 22 negative fluxes were recorded for CO_2_ and CH_4_, respectively. These mainly occurred at the last sampling points (106 and 111 days) where the concentrations were closer to ambient. Figure 7a, c shows the results for the recorded fluxes (in g/m^2^/day), and Fig. 7b, d shows the modelled trend recognised by the spline terms, for CO_2_ and CH_4_, respectively. The statistical modelling indicated that there was significant curvature over time for both CO_2_ (*χ* ^2^ = 10.1 on 1 df, *p* < 0.001) and CH_4_ (*χ* ^2^ = 162.4 on 1 df, *p* < 0.001), and only for CO_2_ was this significantly different (*χ* ^2^ = 19.05 on 1 df, *p* < 0.001, for the spline term) between chambers located directly at the top (middle) and at NW and SE-facing sides of the apex, meaning they showed different patterns of observed fluxes.

For CO_2_, the estimated autocorrelation between time points was not strong at 0.574 (SE 0.086), suggesting that the pattern of the observed fluxes was closer to random than to that of following serial stepwise changes. There appeared to be two main peaks in CO_2_: an early peak before 20 days and then another just before 40 days (Fig. 7a). The first peak could be associated with the peak in probe concentrations, but the second peak is more difficult to explain. Overall, as for the probe data, positive correlation was found between the CO_2_ flux from the heap and average, minimum and maximum temperature records from the core and top (*r* > 0.5, *p* < 0.001, *F* tests).

The predicted value plot, showing the contribution of the spline terms, exhibits a most prominent flux of CO_2_, from the SE side of the heap at around 10 days, then from the NW at the 40 days, and finally from the SE again at around 85 days (Fig. 7b). Such differences between the specific sides of the apex could be caused by wind. During the course of the experiment, winds predominantly originated from a SW direction (49 %), with occasional slower NW and SE winds. Given the alignment of the heap, zone 1 would have received the majority of the head-on wind, but both sides, although mainly the NW side, would have intercepted some crosswinds (Fig. 6).

Comparing Figs. 3a and 6a, it is possible to seek crude links between the wind direction and CO_2_ fluxes. Hence, one could assume that crosswinds from the NW forced the flux to the opposite side of the heap on days 10–20, i.e. during the first peak which is associated mainly with the SE side. For the second peak (mainly associated with the NW), the wind speed could have been generally low but mainly acting against the SE side. For the latter parts of the experiment, wind can be assumed to have predominantly intercepted the NW side once more, so that there was an increase in SE fluxes again. No clear conclusions can be made from this however, and the overall quality of the flux data is questionable.

Very small fluxes of methane were detected: The greatest emission was 0.002 g CH_4_/m^2^/day (Fig. 7c) compared to 2.25 g CO_2_/m^2^/day (Fig. 7a). The autocorrelation between time points was stronger for CH_4_, at 0.709 (SE 0.041), suggesting that there was more serial pattern in the data. This can be seen in Fig. 7d, in that the spline term suggests one very small and negligible peak in CH_4_ flux between days 37 and 80. However, there were periods of relatively high variation between time points, most noticeably on days 35, 69, 77 and 80 and between days 87 and 104. There were no significant (*p* < 0.05, *F* test) effects of location having accounted for the correlation over time and the overall curvature of the data.

## Discussion

There are some similarities in findings between this and other studies on short rotation woody crops. Firstly, the temperature trend observed in the heap was similar to that observed in previous experiments on SRC willow [7, 16] and poplar [17, 18]. Although it is noted that the observations were taken at 3–4 days, or weekly, rather than daily, this result suggests that the process of heating and cooling occurred due to biological processes taking place in the heap rather than due to ambient heating, for which strong autocorrelation would be expected. The self-heating (mesophilic) phase comprises exothermic and aerobic microbial degradation, which is ‘fuelled’ by readily available sugars and starches [6]. As these represent a relatively small proportion of the biomass [19], this phase is estimated to have only lasted for 4 days in the current study. The results suggest that the heap then entered the thermophilic phase (>40 °C) for about 20 days before declining in temperature. Greater fluctuations in temperature in the top of the heap may be due to the rise of heat from the core and the more rapid heat loss due a smaller mass of chip at the top, and the apparent association of cooling occurring after rainfall events suggests that this is due to the evaporation of water. It was not possible to take MC measurements during the course of the experiment, which would have disturbed the heap. It cannot be determined when the drying occurred, though it is likely that the heating phase provided the thermal energy needed to evaporate water from the chip.

As in two other studies on wood storage heaps [6, 7], CO_2_ was the predominant GHG present in the heaps. The rapid pulse of CO_2_ after heap establishment found here was also comparable with previous work. The patterns suggest that the majority of carbon is lost during the initial heating phase of the heap, where a positive correlation is seen between the heap temperature and CO_2_ concentrations. This provides evidence for a rapid aerobic and exothermic degradation phase as the heap moved from a mesophilic to a thermophilic state. It is possible that drying of the chips creates a less favourable environment for microorganisms to operate, which may also be why there is a decline in CO_2_ concentrations after the temperature has declined. The cooling phase indicates a shift back to mesophilic conditions and hence the decay of more recalcitrant compounds such as cellulose, hemicellulose and lignin, which are mainly decomposed by slow-acting fungi that operate best at lower temperatures [17]. A study examining poplar chips [17] found that dry matter losses plateaued as the heap cooled to around mesophilic temperatures at about 2–3 months after heap establishment, which coincided with the end of the peak in CO_2_ concentrations.

The CH_4_ results from the top probes are novel, and these probes show very different concentrations of CH_4_ compared to side probes, as seen in this and previous studies [6, 7]. It is not clear, however, what the top probes indicate in terms of processes taking place in the heap. It is suggested that CH_4_ can become ‘trapped’ in compost heaps, often being revealed as peaks in emissions of CH_4_ during the first time the heap is turned [20, 21], so it is possible that the top probes indicated such accumulation. It could also be the case that the probes were acting as much longer, thinner chambers, suggesting that it is possible to detect fluxes from the heap if there is sufficient penetration into the core of the heap.

The timing of the rise in CH_4_ concentration suggests that anaerobic conditions developed in the heap after the initial heating phase. Although oxygen concentrations were not recorded in the current study, a similar study found that O_2_ fell as CO_2_ concentrations increased during the initial heating phase [17]. Therefore, this study further supports the finding that there is some production of CH_4_ in willow wood storage heaps when stored on grasslands.

The static flux chambers were not effective in monitoring gas fluxes from the heap as throughout the course of the experiment, a number of flux boxes showed non-linear and fluctuating concentrations of gases, which suggests that leakages occurred. Negative fluxes indicate a drop in gas concentration during the sampling time which either suggests that gases can escape from the chamber during the sampling time or are able to re-enter the heap. This is a complication of using flux chambers on highly porous surfaces, and it is possible that they disturb the chimney effect, meaning that gases escape around the chamber instead of into it [14]. The depth of insertion into the heap was set according to soil sampling methods [13, 22], and it is not clear whether a deeper setting of the boxes would have been better for wood chips.

Overall, the quantities of gases detected in the chambers were minute. Previous studies on wood chip heaps suggest that this is because the concentrations found within the heaps were not sufficient to lead to detection of gases in surface chambers. For example, one study did not detect CH_4_ in the chambers until concentrations recorded at the centre of the heap reached 500 ppm [23]. A study on sawdust heaps found that the CH_4_ concentrations detected in probes were much lower than those measured at the surface using chambers [24]. A study on compost heaps noted that CH_4_ was not detected outside the heap until internal concentrations reached 20,000 ppm [25]. Therefore, with the concentrations observed (max 400 ppm), it is possible that generated CH_4_ was oxidised before migrating from the stack [12] or dissipated from the sides of the heap rather than the chimney.

Integrating the average flux result for the gases gives a loss of 0.61 kg carbon from the heap during the storage period, or 1.2 kg DM of wood chips, which compared to the measured DM loss of 5 to 6 t, is a clear underestimation. This was also found by Anderson et al. [14], who compared the static chamber method with a whole-heap dynamic cover approach in a study of compost heaps. They suggested that, for heaped biomass, flux chambers cannot fully address spatial and temporal variations in fluxes without collecting numerous measurements. This problem and issues of leakage already identified suggest that this is not a suitable method for assessing fluxes from porous or bulky materials. The whole-heap dynamic cover would be an alternative approach but would have curtailed the external environmental aspects of the current investigation.

## Conclusion

After establishing the SRC willow chip heap, temperatures over 60 °C were observed after 10 days. This is consistent with current studies on the storage of willow and poplar chips. Probes embedded in the stack showed an increase of CO_2_, peaking at around 70,000 ppm from vertical probes and 20,000 ppm from horizontal probes. This rise correlated with the initial rise in temperature. As CO_2_ dropped, a small peak (400 pm) was observed in CH_4_, though only from probes orientated vertically in the top of the heap.

Based on both the probe concentration and chamber flux results, the emission of CH_4_ seemed to occur as a single peak with a rise and fall over a long period, rather than a continuous emission as suggested elsewhere [26, 27]. Sampling fugitive GHG emissions using chamber boxes could be prone to leakage or could disrupt the chimney effect observed in wood chip heaps, allowing gas to escape around the chamber. However, there appears to be some correspondence between the observed peaks in flux and the results from the top probes. For example, the first peak of CO_2_ around day 20 and the peak of methane between days 50 and 70 are consistent for the two techniques. This suggests that the chamber flux measurements were not entirely inaccurate. A solution may be to use larger chambers that penetrate deeper into the heap or to cover the entire heap during the sampling periods.

## Electronic supplementary material

Supplementary data 1 Trends recognised by the spline terms for concentrations of a) CO_2_ and b) CH_4_ in the side and top probes. (PNG 190 kb)
