# Supplementary figures and images for: Testing the Use of Static Chamber Boxes to Monitor Greenhouse Gas Emissions from Wood Chip Storage Heaps
Source: Bioenergy Res. 2016 Nov 9;10(2):353–62. doi: 10.1007/s12155-016-9800-9 (PMC7010367; doi:10.1007/s12155-016-9800-9)

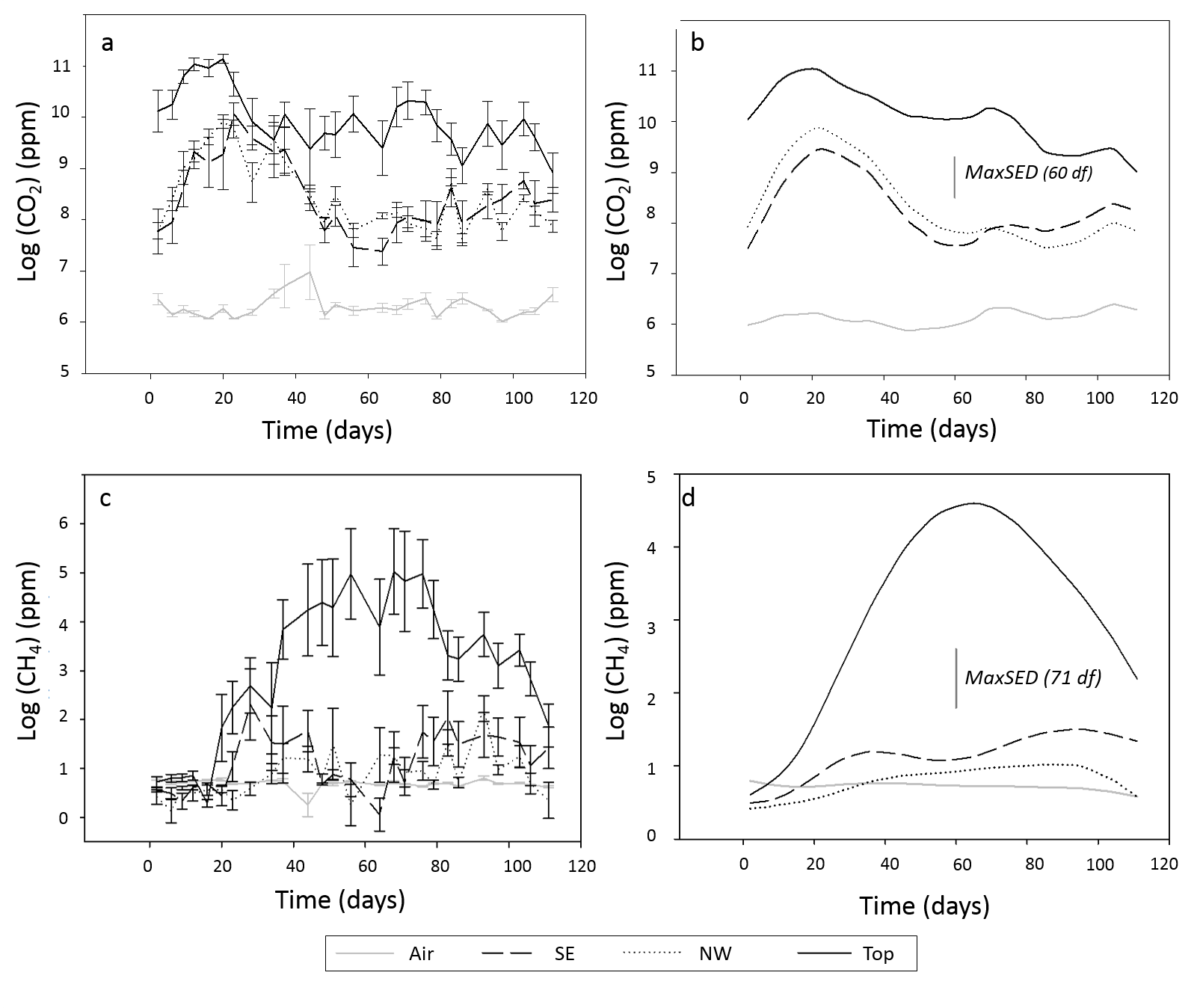

Supplement: Supplementary file 1 — Trends recognised by the spline terms for concentrations of a) CO2 and b) CH4 in the side and top probes. (PNG 190 kb) [file 12155_2016_9800_Fig8_ESM.png]
